# Stem Cell Markers CXCR-4 and CD133 Predict Aggressive Phenotype and Their Double Positivity Indicates Poor Prognosis of Oral Squamous Cell Carcinoma

**DOI:** 10.3390/cancers13235895

**Published:** 2021-11-23

**Authors:** Ravindran Caspa Gokulan, Halagowder Devaraj

**Affiliations:** Unit of Biochemistry, Department of Zoology, Guindy Campus, University of Madras, Chennai 600085, Tamil Nadu, India; hdrajam@unom.ac.in

**Keywords:** CXCR-4, oral cancer, cancer stem cells, CD133, PKC-δ

## Abstract

**Simple Summary:**

Oral cancer is one of the most frequent malignancies in the world, with a poor prognosis. The absence of accurate biomarkers for predicting oral cancer progression is the primary cause of treatment failures. Multiple studies have shown that cancer stem cells play a critical role in tumor growth and chemo resistance. We uncovered, for the first time, the importance of combinatorial expression of stem cell related molecules CXCR-4 and CD-133 as possible biomarkers to predict poor prognosis of oral squamous cell carcinoma. The findings will aid in the identification of high-risk cases in order to provide appropriate therapy.

**Abstract:**

The activation of the SDF-1/CXCR-4 pathway is crucial for the invasion and metastasis of oral cancer cells. The CXCR-4 positive cells possess stem cell characteristics and express the cancer stem cell marker, CD133, in tumors of colon and pancreas. Despite several studies, the co-expression of CXCR-4 and CD133 and its significance is still largely unknown in oral cancer. Therefore, we aimed to investigate the impact of CXCR-4 and CD133 double positivity in the prognosis of oral cancer. The significance of PKC-δ, one of the key signaling molecules that regulates CXCR-4, was also analyzed. Immunohistochemistry and double immunofluorescence was used to investigate the co-localization of CXCR-4, PKC-δ and CD133 in the human tissues and cell lines of oral squamous cell carcinoma. The expression of CXCR-4, PKC-δ and CD133 were found to be higher in poorly differentiated and lymph node metastasis-positive cases. Interestingly, CXCR-4 positive cells showed positive staining for PKC-δ and CD133 in oral cancer tissue and cell lines. Moreover, CXCR-4+/CD133+ and CXCR-4+/PKC-δ+ double positive cases have the worst survival. We discovered, for the first time, that patients with expression of both CXCR-4 and CD133 have a lower survival rate, and CXCR-4+/CD133+, as well as CXCR-4+/PKC-δ+ double positivity, can be utilized to predict poor prognosis. CXCR-4, PKC-δ and CD133 might regulate aggressiveness and invasion of oral cancer cells.

## 1. Introduction

Cancer is a complex heterogeneous disease that develops as a consequence of genetic and epigenetic alterations. The sequential events begin in the precancerous stage and control tumor growth. Oncogene and tumor suppressor gene alterations were highly crucial for the development of cancer [1]. Oral cancer ranks sixth among the most common cancers in the world and the 5 year survival rate has not been substantially increased yet [2]. The failure of advanced treatments may be due to the lack of specific markers to predict the outcome and to identify high risk cases. Several reports have pointed out the significance of using a combination of functionally related markers to predict poor prognosis of oral cancer. The re-expression of stemness markers such as Oct-4, c-Myc, Nanog and CD44 in oral cancer tissues and their association with recurrence provides a platform for Scientists to explore their significance in predicting poor prognosis [3,4]. In this study, we have investigated the expression pattern of CXCR4, PKC-δ and CD133 in oral squamous cell carcinoma. The CXCR-4+/CD133+ and CXCR-4+/PKC-δ+ double positivity was also analyzed to predict poor prognosis.

According to the cancer stem cell hypothesis, tumor tissue contains a subset of cancer stem cells (CSCs) that control tumor growth [5]. The epithelial-to-mesenchymal transition (EMT) is associated with invasion and metastasis. It also partly generates cells with stem-like characteristics that showed resistance to chemotherapeutic drugs and radiation [6]. They have been found to be involved in the migration and metastasis of cancer cells. C-X-C chemokine receptor type 4 (CXCR-4) is present in stem cells of skeletal muscle, neural, retinal and liver tissues [7]. CXCR-4 is activated by the binding of its ligand Stromal derived factor-1 (SDF-1) and the CXCR-4-positive cells tend to migrate towards the SDF-1 gradient [8,9]. CXCR-4 is a G-protein coupled receptor which exerts its function through several signaling cascades including PI3K, MAPK, AKT, ERK and NFĸB [10,11,12]. SDF-1/CXCR-4 signaling regulates proliferation, angiogenesis, chemotaxis and metastasis of cancer cells. Higher levels of CXCR-4 expression have been found in different types of cancer such as cancers of the lung, kidney, liver, colon, oral, esophagus, pancreas and brain [13]. In addition to oral cancer tissues, CXCR-4 expression has also been identified in oral precancerous lesions [14]. CXCR-4 inhibition causes oral cancer cells to lose their ability to metastasize and proliferate. [15]. SDF-1/CXCR-4 was found to be involved in regulating the lymph node metastasis of tumors [16]. SDF-1 and CXCR4 polymorphisms are linked to oral and pharyngeal squamous cell carcinoma susceptibility [17]. Interestingly, TGF-β1-induced EMT of oral carcinoma cells has found to be accompanied by an increase in CXCR-4 expression [18]. CXCR4 increases the resistance of human tongue squamous cell carcinoma cells to cisplatin [19].

In hematopoietic progenitor cells, cAMP-induced PKC-ζ positively regulates the expression of CXCR-4 [20]. The hepatocyte growth factor (HGF) has also been found to regulate the CXCR-4 expression by activating PKC-ζ in breast cancer cells [21]. In addition to PKC-ζ, PKC-δ is a serine/threonine kinase that regulates the proliferation and survival of cancer cells [22]. The cancer stem cell-like cells require PKC-δ to exert their oncogenic function. The inhibition of PKC-δ by small molecules or siRNA attenuates proliferation and survival of both non-CSC and CSC populations [23]. The CXCR-4 and CD133 positivity was found to have prognostic value in colon cancer [24].

CD133 (prominin-1) is a transmembrane glycoprotein initially identified in neuroepithelial and hematopoietic stem cells [25,26]. CD133 expression has been observed in several tumors and it is being used to isolate cancer stem cells from glioma, colon and oral cancers [27,28,29]. The expression of CD133 was found to be gradually increased from normal to dysplasia to oral squamous cell carcinoma. In addition, CD133-positive oral cancer stem-like cells express the pluripotent markers like Oct-4, c-Kit and Nanog [29,30]. CD133^+^ oral cancer cells were found to have chemoresistance and the knockdown of CD133 inhibits proliferation [31,32]. CD133+/CXCR-4+ cancer stem cells were found to play a pivotal role in metastasis of colorectal cancer and the EMT makes them more migratory [33]. The CD133+/CXCR-4+ subpopulation of cancer cells was found to possess migratory potential and induce metastasis of pancreatic cancer [34]. In oral squamous cell carcinoma, side population cells with CSC features existed, and silencing CD133 had a significant therapeutic potential in increasing chemotherapy sensitivity by eliminating CSCs [35].

Although CXCR-4 and CD133 have been examined for their expression patterns and prognostic value in oral cancer, their association has yet to be uncovered. Furthermore, in oral squamous cell carcinoma, the expression pattern of PKC-δ and its relationship to clinicopathological variables has not been fully examined. On the other hand, the significance of CXCR-4/CD133 and CXCR-4/PKC-δ double positivity is untouched in oral squamous cell carcinoma. Taken together, the aim of our study is to investigate the expression pattern of CXCR-4, PKC-δ and CD133 in oral cancer tissues and cell line. We also studied their correlation with clinicopathological factors and the possibility of using CXCR-4/CD133 as well as CXCR-4/PKC-δ double positivity to predict poor prognosis in oral cancer.

## 2. Materials & Methods

### 2.1. Tissues & Cell Line

The tissues were obtained from the de-identified tissue blocks of 51 patients referred to the Government Royapettah Hospital in Chennai, aged 40 to 70, with a mean age of 58.7. The average follow-up length was 38.7 months, with a range of 14 to 59 months. The sample’s histopathological characteristics were evaluated using the criteria outlined by Pindborg et al. [36]. After being fixed in 10% buffered formalin, the tissues were processed for paraffin embedding. For histopathological and immunohistochemical studies, 4 µm tissue sections were used. With clearance from the Directorate of Medical Education, Government of Tamil Nadu, India, the study was approved by the Hospital Medical Board (No. 14769/E1/2008-1). The written consent was waived due to the de-identified nature of the samples. The H314 cell-bearing slides (derived from a poorly differentiated tumor of the floor of the mouth) were received as a gift from Dr. Angela Hague (Department of Oral and Dental Science, University of Bristol, UK). The CAL27 cell line derived from squamous cell carcinoma of human tongue was obtained as a gift from Dr. Karl Kingsley (School of Dental Medicine, University of Nevada, Las Vegas, NV, USA).

### 2.2. Immunohistochemistry

The samples were deparaffinized in xylene and processed through a graded series of alcohol. The sections were then microwaved in 0.1 M sodium citrate buffer (pH 6.0) for antigen retrieval. The slides were cooled at room temperature and the endogenous hydrogen peroxidase activity was inhibited with 3% hydrogen peroxide for 10 min. The non-specific binding sites were blocked for 30 min at room temperature with 0.3% Bovine Serum Albumin (BSA). The sections were then incubated with Mouse monoclonal anti-human CXCR-4 antibody (a kind gift from Dr. Le Bousse, Paris-Sud University, France; dilution—1:200), goat Polyclonal anti-CD133 (Santa Cruz Biotechnology, Santa Cruz, CA, USA. Headquarters: Dallas, TX, USA; dilution—1:200) and rabbit polyclonal anti-PKC-δ (Oncogene-Transduction Laboratories, Sanjose, California, USA. (now a part of BD biosciences); dilution—1:100) overnight at 4 °C. The secondary antibodies were horseradish peroxidase conjugated and used to stain the slides (Invitrogen, Waltham, MA, USA). The antibody binding was then identified with chromogen 3, 3′ diaminobenzidine as a substrate. The sections were examined after counter-staining with hematoxylin. For each staining, negative controls without the primary antibody were included. The staining was assessed at 200× magnification in three to five random fields for scoring. The slides were evaluated independently by two observers without information on the patient’s clinicopathological data. The scoring was done as described previously [30]: 0 (negative), staining was not observed or staining observed in less <5% of cells; 1+ (mild), staining observed in 5–15% of cells; 2+ (moderate), staining observed in 16–25% of cells; 3+ (intense), staining in >25% of cells. The staining was then grouped as Negative (0 and 1+ categories) and Positive (includes 2+ and 3+ categories). The stained cell percentage refers to cancer cells. The slides were re-evaluated by the two observers in the event of dispute, and a consensus was established after discussion.

### 2.3. Immunofluorescence

Immunofluorescence analysis has been carried out using our previous protocol [37]. After being deparaffinized and rehydrated, the cells were fixed for 5 min. The permeabilization was done with 0.1% Triton X-100 and the blocking was done using 1% BSA for 30 min. The slides were then incubated with primary antibody for overnight at 4 °C, followed by incubation for 2 h with Alexa Fluor 594 (red) conjugated secondary antibody for CXCR-4 and FITC conjugated secondary antibody for PKC-δ and CD133 after being washed with PBS. The slides were then mounted and analyzed using a confocal fluorescence microscope (Leica TCS SP2, Leica Microsystems, Wetzlar, Germany).

### 2.4. Statistical Analysis

The correlation between clinicopathological features and protein expression was explored using the Chi square test. For 2 × 2 tables, Fisher’s exact test was used. The association among CXCR-4, PKC-δ, and CD133 expression was calculated by Spearman’s correlation analysis. The Kaplan–Meier method was used to generate the overall and disease-free survival curves, and the log-rank test was employed to compare the two curves. The time between therapy and recurrence or metastasis or disease-related mortality was measured to determine disease-free survival. The cases were censored either at the time of death for patients who died during the study or at the time of the last examination for patients who did not receive a follow-up examination. The Cox proportional hazards regression model was used to perform the Univariant and Multivariant survival analyses. The survival analysis was done using SPSS software (SPSS for windows 14.0, SPSS Inc., Chicago, IL, USA) and the statistical analyses were done with Acastat Statistical software, version 6.1 (Acastat Software, Winter Garden, Florida, USA). For all statistical studies, a *p* < 0.05 was considered significant. Multiple tests were used without adjustments due to the exploratory nature of the study.

## 3. Results

### 3.1. CXCR4

**Figure 1:**
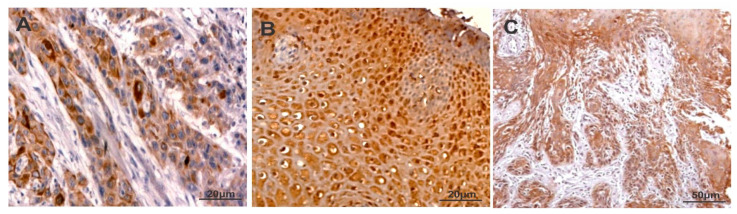
Immunostaining of CXCR-4 (**A**), PKC-δ (**B**) and CD133 (**C**) in oral squamous cell carcinoma.

The staining of CXCR-4 was intense and predominantly found in the cytoplasm of oral carcinoma cells (Figure 1 and Supplementary Figure S1). The staining score of CXCR4 in oral cancer tissues was provided in Table 1. The CXCR-4 expression was found to be higher in cases with lymph node metastasis compared with cases without lymph node metastasis. A statistically significant difference was found between CXCR4 and grades of oral squamous cell carcinoma (*p* < 0.02). However, a significant correlation was not reached between the expression levels of CXCR-4 and stage, age, location of the tumor as well as sex (Table 2).

### 3.2. PKC-δ

The expression of PKC-δ was predominantly observed in the cytoplasm and nucleus of oral carcinoma cells (Figure 1 and Supplementary Figure S1). Table 1 summarizes the staining scores of PKC-δ in oral squamous cell carcinoma tissues. Higher levels of PKC-δ expression were found in stage III–IV than stage I–II of oral squamous cell carcinoma (*p* < 0.02). In addition, cases with lymph node metastasis showed increased PKC-δ expression than lymph node metastasis-negative cases (*p* < 0.001). Moreover, the expression of PKC-δ was not associated with stage, age, location of tumor and sex (Table 2).

### 3.3. CD133

The expression of CD133 was mainly localized in the cytoplasm of oral carcinoma cells. However, some oral carcinoma cells showed both membranous and cytoplasmic expression of CD133 (Figure 1 and Supplementary Figure S1). The staining score of CD133 in oral cancer tissues is provided in Table 1. The expression of CD133 was significantly higher in stage III–IV when compared with stage I–II (*p* < 0.001). The poorly differentiated tumors showed higher levels of CD133 expression than moderate and well differentiated tumors. The CD133 expression had no significant association with stage, age, location of the tumor as well as sex. Moreover, The CD133 expression was found to be higher in cases with lymph node metastasis compared to cases without lymph node metastasis. (*p* < 0.008) (Table 2).

### 3.4. Correlation of CXCR-4 Expression with PKC-δ and CD133 in Oral Squamous Cell Carcinoma

**Figure 2:**
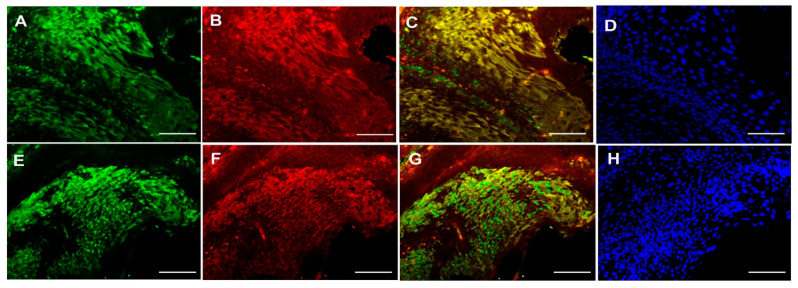
Double Immunofluorescence analysis of CXCR-4 with PKC-δ and CD133 in human oral carcinoma tissues. Upper panel: CXCR4 (**A**), PKC-δ (**B**), CXCR-4 and PKC-δ co-localization (**C**) and DAPI (**D**) staining in oral cancer tissue. Lower panel: CXCR4 (**E**), CD133 (**F**), CXCR-4 and CD133 co-localization (**G**) and DAPI (**H**) staining in oral cancer tissue (Scale bar—20 μm).

**Figure 3:**
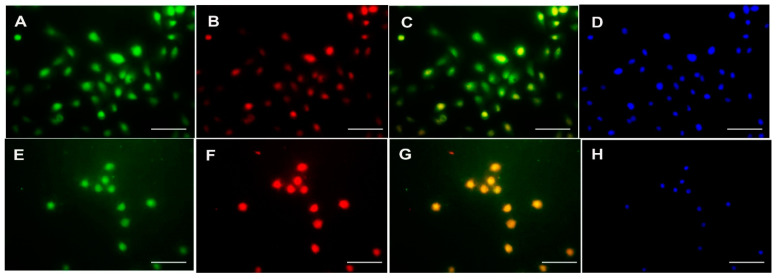
Double Immunofluorescence analysis of CXCR-4 with PKC-δ and CD133 in human esophageal cancer cell line H314. Upper panel: CXCR4 (**A**), PKC-δ (**B**), CXCR-4 and PKC-δ co-localization (**C**) and DAPI (**D**) staining in H314 cells. Lower panel: CXCR4 (**E**), CD133 (**F**), CXCR-4 and CD133 co-localization (**G**) and DAPI (**H**) staining in H314 cells (Scale bar—20 μm).

The correlation between the expression of PKC-δ, CD133 and CXCR-4 was investigated using Spearman’s correlation analysis (Table 3). Interestingly, no significant association was found between the expression of PKC-δ, CD133 and CXCR-4 in oral cancer (*p* > 0.05). On the other hand, the co-localization of CXCR-4 with PKC-δ or CD133 was studied using immunofluorescence in the tissues and cell line of oral cancer. The co-expression of CXCR-4 and PKC-δ has been found in some cells of oral squamous cell carcinoma. Notably, the CXCR-4-positive cells were found to express CD133 in human oral cancer tissues (Figure 2) and cell lines (Figure 3 and Supplementary Figure S2).

### 3.5. Survival Analysis

**Figure 4:**
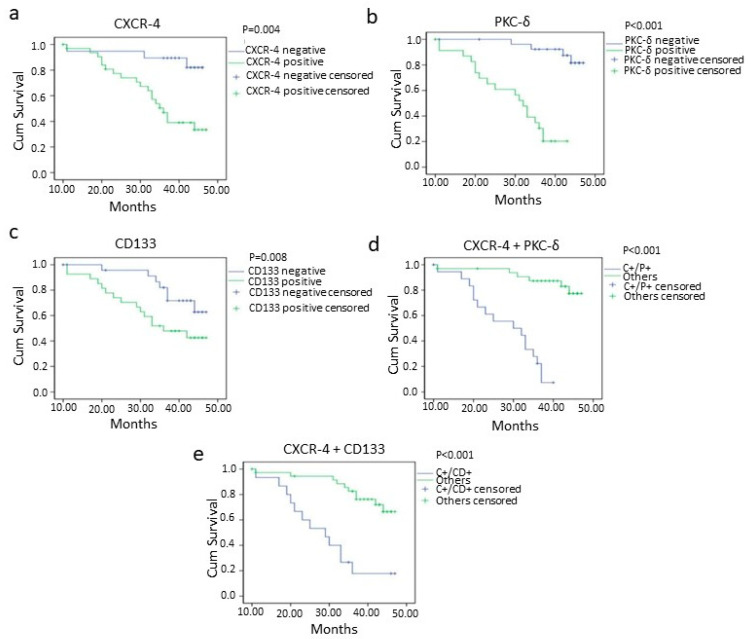
Overall survival of oral squamous cell carcinoma patients with CXCR-4 (*p* = 0.004) (**a**), PKC-δ (*p* = <0.001) (**b**), CD133 (*p* = 0.008) (**c**), CXCR-4+PKC-δ (double positive; *p* = <0.001) (**d**) and CXCR-4 + CD133 (double positive; *p* = <0.001) (**e**) positivity analyzed using the Kaplan–Meier method. negative= 0 and 1+ categories, positive = 2+ and 3+ categories, C+/P+ = CXCR-4 and PKC-δ double positive cases, C^+^/CD^+^ = CXCR-4 and CD133 double positive cases.

**Figure 5:**
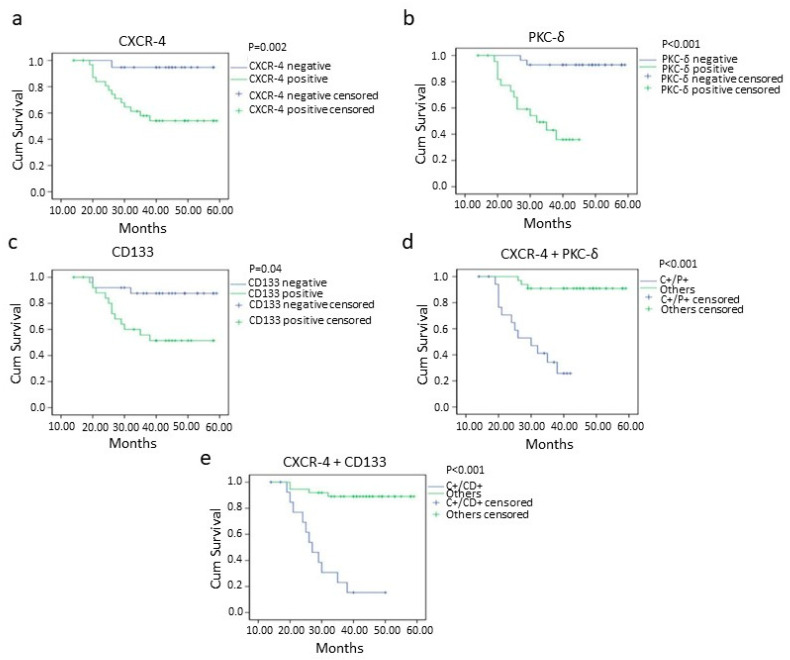
Disease-free survival of oral squamous cell carcinoma patients with CXCR-4 (*p* = 0.002) (**a**), PKC-δ (*p* = <0.001) (**b**), CD133 (*p* = 0.04) (**c**), CXCR-4+PKC-δ (double positive; *p* = <0.001) (**d**) and CXCR-4 + CD133 (double positive; *p* = <0.001) (**e**) positivity analyzed by Kaplan–Meier method. Key: − = 0 and 1+ categories, + = 2+ and 3+ categories, LN = Lymph node, C+/P+ = CXCR-4 and PKC-δ double positive cases, C+/CD+ = CXCR-4 and CD133 double positive cases.

The worst overall and disease-free survival was found in CXCR-4-, PKC-δ- and CD133-positive cases than negative cases (Figure 4 and Figure 5). The oral squamous cell carcinoma cases with CXCR-4/PKC-δ, CXCR-4/CD133 and CD133/PKC-δ double positivity showed poor survival rates (Figure 4 and Figure 5). Markedly, higher clinical stage and lymph node metastasis were significantly associated with poorer survival in oral cancer (Table 4; Supplementary Figures S3 and S4).

The prognostic significance of CXCR-4, PKC-δ and CD133 was explored using Cox Proportional Hazards Regression model. The univariate Cox proportional hazards method demonstrated the prognostic value of lymph node metastasis, stage, CXCR-4, PKC-δ and CD133 in oral squamous cell carcinoma (Table 5). Interestingly, in multivariate analysis, CXCR-4, PKC-δ and CD133 were found to be an independent prognostic factor in disease-free conditions (Table 6).

## 4. Discussion

The cancer cells which escape chemo and radiation therapies were supposed to be responsible for the recurrence of tumors. Several studies have reported the presence of a subpopulation of cancer cells with stem cell characteristics and termed as cancer stem cells. These cells were found to have the ability to exclude anti-tumor drugs and showed resistance to advanced therapies [38]. The oral cancer patients with positive expression of the stem cell markers such as Oct-4, CD44 and c-Myc showed resistance to radio-chemo therapies. In addition, the stem cell markers were found to have a prognostic value and predict poor survival [4]. EMT causes an increase in cancer stem cell phenotypes, which are characterized by markers such as Oct-4, Nanog, CD44, and CD133 [6]. In this study, we have investigated the importance of CXCR-4 and CD133 or PKC-δ double positivity in the prognosis of oral squamous cell carcinoma.

CXCR-4 is a chemokine receptor that regulates chemotaxis, proliferation and invasion of oral cancer cells [39,40,41]. SDF-1/CXCR-4 signaling exerts its function through activating intermediate molecules like NFĸB, AKT, MMPs, ERK and MAPK in cancer tissues [10,11,12]. Several studies have reported the upregulation of CXCR-4 in oral cancer cells when compared with normal epithelium [14,40,42]. In the present study, the cytoplasmic expression of CXCR-4 was predominantly found as mentioned in the previous report of Xia et al. [14]. In addition, CXCR-4 expression was not associated with stage, gender, age and location of the tumor. The expression of CXCR-4 was found to be associated with the grade of esophageal squamous cell carcinoma [43]. In this study, the poorly differentiated tumors have higher levels of expression of CXCR-4 than moderate and well-differentiated tumors. As reported by Lee et al. [42], the expression levels of CXCR-4 showed a significant difference between lymph node metastasis-positive and lymph node metastasis-negative cases. Particularly, the expression of CXCR-4 has been found to be higher in lymph node metastasis-positive cases when compared to lymph node metastasis-negative cases. These results indicate that the CXCR-4 might control the differentiation and metastasis of oral cancer cells.

The expression of CXCR-4 has been increased upon activation of PKC-ζ in hematopoietic progenitor and cancer cells [20,21]. Therefore, we are interested to investigate the expression pattern of PKC-δ and its correlation with clinicopathological factors in oral squamous cell carcinoma. In our present study, the expression of PKC-δ was predominantly found in the cytoplasm of oral cancer cells. A statistically significant difference was found in PKC-δ expression among different stage groups. The difference in PKC-δ expression was also noticed between lymph node metastasis-positive and -negative groups. However, no significant difference was found between age, sex, histological grade, location of tumors and PKC-δ expression. These results suggest that the PKC-δ might regulate the aggressiveness and invasion of oral cancer cells.

The CXCR-4 signaling pathway regulates migration and metastasis of oral cancer cells [44]. The CXCR-4+/CD133+ cancer cell population was increased during EMT and possesses the characteristics of stem cells [33,34]. CD133 is a cancer stem cell marker expressed in oral cancer tissues and oral cancer stem-like cells. The CD133^+^ cells were found to have chemoresistance and are positive for Oct-4, Nanog and c-Kit [29]. In the present study, the cytoplasmic expression of CD133 was observed in oral squamous cell carcinoma tissue which was in agreement with the previous report of Chiou et al. [29]. A statistically significant difference was found between lymph node metastasis positivity and CD133 expression. As in line with our previous report [45], stage III/IV have high CD133 expression compared with stage I/II. Additionally, the expression of CD133 showed a significant difference between different grades of oral squamous cell carcinoma. There is no significant difference between CD133 expression and location of the tumor, gender as well as age of oral cancer patients. These results suggest that the CD133 might regulate proliferation and invasion of oral cancer cells.

Recently, the CD133+/CXCR-4+ populations of cancer stem cells were found to be the possible migratory cells and the co-expression of CXCR-4 and CD133 had prognostic value in colon cancer [24,33]. In this study, the co-localization of CXCR-4/CD133 and CXCR-4/PKC-δ was found in oral carcinoma tissues and cell lines. The result indicates that they might have a functional relationship in oral cancer. However, it needs further investigation. In line with the findings of Lee et al. [42], the oral squamous cell carcinoma patients with high CXCR-4 expression have less survival. Similar to CXCR-4, less survival time was observed in patients with high expression of PKC-δ. On the other hand, CD133 expression has been significantly correlated with survival in overall and disease-free survival conditions, which was in agreement with the report in esophageal cancer [46]. Moreover, CXCR-4/CD133, CXCR-4/PKC-δ and CD133/PKC-δ double positivity were highly correlated with worse survival in oral cancer as calculated by Kaplan–Meier analysis. These results suggest that CXCR-4/CD133, CXCR-4/PKC-δ and CD133/PKC-δ double positivity might help to recognize patients with high chance for effective treatment. In the present study, the univariate analysis revealed the prognostic significance of CXCR-4, PKC-δ and CD133 in oral squamous cell carcinoma. However, the multivariate analysis explored the significance of using CXCR-4, PKC-δ and CD133 as independent prognosticators in the disease-free survival condition.

## 5. Conclusions

Increased expression of CXCR-4, PKC-δ and CD133 indicates their role in the progression of oral cancer. CXCR-4, PKC-δ, and CD133, in particular, may control the aggressive phenotype and invasion of oral cancer cells. The co-expression of CXCR-4/PKC-δ and CXCR-4/CD133 in oral cancer tissues and cell lines suggest that they may have a functional interaction in oral cancer cells. For the first time, we found that the CXCR-4+/CD133+ and CXCR-4+/PKC-δ+ double positivity might help to recognize patients with high chance for effective treatment. CXCR-4, PKC-δ and CD133 were found to be independent prognosticators of disease-free survival. Further studies on the functional interaction and molecular targeting of CXCR-4, PKC-δ and CD133 could pave a way for novel therapeutic interventions.

## Figures and Tables

**Table 1:** Immunodetection of CXCR-4, PKC-δ and CD133 in oral squamous cell carcinoma.

Protein	No. of Cases	0	1+	2+	3+
CXCR-4	51	11	7	15	18
PKC-δ	51	13	8	20	10
CD133	51	9	13	18	11

Key: 0 (negative), no staining or <5% positive cells; 1+ (mild), 5–15% positive cells; 2+ (moderate), 16–25% positive cells and 3+ (intense), >25% positive cells.

**Table 2:** Association between CXCR4, PKC-δ and CD133 and clinicopathological factors.

		CXCR-4			PKC-δ			CD133		
		Negative (18)	Positive (33)	*p* Value	Negative (21)	Positive (30)	*p* Value	Negative (22)	Positive (29)	*p* Value
No. of Patients										
Age										
≤60	26	12	14	*p* < 0.14	10	16	*p* < 0.77	8	18	*p* < 0.09
>60	25	6	19		11	14		14	11	
Gender										
Male	29	11	18	*p* < 0.77	15	14	*p* < 0.09	14	15	*p* < 0.56
Female	22	7	15		6	16		8	14	
Stage										
I–II	24	9	15	*p* < 0.78	14	10	*p* < 0.02 *	16	8	*p* < 0.001 *
III–IV	27	9	18		7	20		6	21	
Lymph node Metastasis										
Negative	23	15	8	*p* < 0.001 **	16	7	*p* < 0.001 **	15	8	*p* < 0.005 **
Positive	28	3	25		5	23		7	21	
Histological Grade										
Well Differentiated	8	6	2	*p* < 0.02 *	6	2	*p* < 0.10	7	1	*p* < 0.008 **
Moderately Differentiated	19	7	12		7	12		9	10	
Poorly Differentiated	24	5	19		8	16		6	18	
Location of the Tumours										
Tongue	15	8	7	*p* < 0.42	7	8	*p* < 0.54	6	9	*p* < 0.56
Buccal	9	3	6		5	4		4	5	
Palate	8	3	5		4	4		4	4	
Gingiva	10	2	8		3	7		6	4	
Floor of Mouth	9	2	7		2	7		2	7	

Negative (0 and 1+ categories) and Positive (2+ and 3+ categories). * Statistically significant, ** Statistically highly significant.

**Table 3:** Association among CXCR-4, PKC-δ and CD133 expression.

Expression of CXCR-4		Expression of PKC-δ				Expression of CD133			
(No. of Patients = 51)		0 (13)	1+ (8)	2+ (10)	3+ (20)	0 (9)	1+ (13)	2+ (11)	3+ (18)
0	11	9	2	0	0	9	2	0	0
1+	7	3	3	1	0	0	5	2	0
2+	15	1	3	2	9	0	6	3	6
3+	18	0	0	7	11 ^a^	0	0	3	12 ^b^

Key: 0 (negative), staining was not observed or staining observed in less <5% of cells; 1+ (mild), staining observed in 5–15% of cells; 2+ (moderate), staining observed in 16–25% of cells; 3+ (intense), staining in >25% of cells. ^a^ = No Significant association between CXCR-4 and PKC-δ expression levels (*p* > 0.05). ^b^ = Significant association between CXCR-4 and CD133 expression levels (*p* > 0.05).

**Table 4:** Survival analysis of clinicopathological variables, CXCR-4, PKC-δ and CD133.

	Variables	Overall Survival	Disease-Free Survival
		*p* Value	*p* Value
1	Age	0.30	0.42
2	Sex	0.78	0.52
3	Stage (I,II/III,IV)	0.0008 **	0.03 *
4	Grade (Well/Moderate/Poor)	0.38	0.53
5	LN Metastasis (+/−)	<0.001 **	0.01 *
6	CXCR-4 (+/−)	0.004 **	0.002 **
7	PKC-δ (+/−)	<0.001 **	<0.001 **
8	CD133 (+/−)	0.008 *	0.04
9	CXCR-4 + PKC-δ (C^+^/P^+^ vs. others)	<0.001 **	<0.001 **
10	CXCR-4 + CD133 (C^+^/CD^+^ vs. others)	<0.001 **	<0.001 **
11	PKC- + CD133 (P^+^/CD^+^ vs. others)	<0.001 **	<0.001 **

− = 0 and 1+ categories, + = 2+ and 3+ categories, LN = Lymph node, C+/P+ = CXCR-4 and PKC-δ double positive cases, C+/CD+ = CXCR-4 and CD133 double positive cases, P+/CD+ = PKC-δ and CD133 double positive cases. * Statistically significant, ** Statistically highly significant.

**Table 5:** Cox univariate proportional hazards regression analysis.

	Variables	Overall Survival			Disease-Free Survival		
		*p* Value	Hazards Ratio	95% CI	*p* Value	Hazards Ratio	95% CI
1	Stage (I,II/III,IV)	0.005 **	8.354	1.877–37.180	0.04 *	2.529	1.028–6.220
2	Grade (Well/Moderate/Poor)	0.394	0.627	0.214–1.836	0.479	0.734	0.312–1.727
3	LN Metastasis(+/−)	0.046 *	10.895	1.072–55.034	0.014 *	3.292	1.274–8.503
4	CXCR-4 (+/−)	0.023 *	10.567	1.388–80.442	0.006 **	5.612	1.651–19.078
5	PKC-δ (+/−)	0.001 **	12.682	2.833–56.771	<0.001 **	14.473	4.034–51.927
6	CD133 (+/−)	0.01 *	4.688	1.321–16.640	0.049 *	2.440	0.993–5.998

− = 0 and 1^+^ categories, + = 2^+^ and 3^+^ categories, LN = Lymph node. * Statistically significant, ** Statistically highly significant.

**Table 6:** Cox multivariate proportional hazards regression analysis.

	Variables	Overall Survival			Disease-Free Survival		
		*p* Value	Hazards Ratio	95% CI	*p* Value	Hazards Ratio	95% CI
1	CXCR-4	0.226	3.645	0.448–29.628	0.029 *	4.652	1.172–18.471
2	PKC-δ	0.107	3.589	0.757–17.004	0.001 **	16.173	3.332–78.496
3	CD133	0.282	2.109	0.541–8.217	0.023 *	3.801	1.202–12.013

* Statistically significant, ** Statistically highly significant.

## Data Availability

All the datasets of this study are presented within the article and as supplementary information. The data can be shared upon reasonable request.

## Supplementary Materials

The following are available online at https://www.mdpi.com/article/10.3390/cancers13235895/s1, Figure S1: Representative images of Immunostaining of CXCR-4, PKC-δ and CD133 in oral squamous cell carcinoma tissues. Figure S2: Double Immunofluorescence analysis of CXCR-4 with PKC-δ and CD133 in human esophageal cancer cell line CAL27. Figure S3: Overall survival of patients with oral squamous cell carcinoma according to Stage and Lymph node metastasis. Figure S4: Disease-free survival of patients with oral squamous cell carcinoma according to Stage and Lymph node metastasis.

## References

[1] Levine A.J., Puzio-Kuter A.M. (2010). The control of the metabolic switch in cancers by oncogenes and tumor suppressor genes. Science.

[2] Warnakulasuriya S. (2009). Global epidemiology of oral and oropharyngeal cancer. Oral Oncol..

[3] Gokulan R., Halagowder D. (2014). Expression pattern of Notch intracellular domain (NICD) and Hes-1 in preneoplastic and neoplastic human oral squamous epithelium: Their correlation with c-Myc, clinicopathological factors and prognosis in Oral cancer. Med. Oncol..

[4] Sawant S., Gokulan R., Dongre H., Vaidya M., Chaukar D., Prabhash K., Ingle A., Joshi S., Dange P., Joshi S. (2016). Prognostic role of Oct4, CD44 and c-Myc in radio-chemo-resistant oral cancer patients and their tumourigenic potential in immunodeficient mice. Clin. Oral Investig..

[5] Reya T., Morrison S.J., Clarke M.F., Weissman I.L. (2001). Stem cells, cancer, and cancer stem cells. Nature.

[6] Singh A., Settleman J. (2010). EMT, cancer stem cells and drug resistance: An emerging axis of evil in the war on cancer. Oncogene.

[7] Kucia M., Ratajczak J., Ratajczak M.Z. (2005). Bone marrow as a source of circulating CXCR4^+^ tissue-committed stem cells. Biol. Cell.

[8] Zou Y.R., Kottmann A.H., Kuroda M., Taniuchi I., Littman D.R. (1998). Function of the chemokine receptor CXCR4 in haematopoiesis and in cerebellar development. Nature.

[9] Kucia M., Reca R., Miekus K., Wanzeck J., Wojakowski W., Janowska-Wieczorek A., Ratajczak J., Ratajczak M.Z. (2005). Trafficking of normal stem cells and metastasis of cancer stem cells involve similar mechanisms: Pivotal role of the SDF-1-CXCR4 axis. Stem Cells.

[10] Tang C.H., Chuang J.Y., Fong Y.C., Maa M.C., Way T.D., Hung C.H. (2008). Bone-derived SDF-1 stimulates IL-6 release via CXCR4, ERK and NF-kappaB pathways and promotes osteoclastogenesis in human oral cancer cells. Carcinogenesis.

[11] Tan C.T., Chu C.Y., Lu Y.C., Chang C.C., Lin B.R., Wu H.H., Liu H.L., Cha S.T., Prakash E., Ko J.Y. (2008). CXCL12/CXCR4 promotes laryngeal and hypopharyngeal squamous cell carcinoma metastasis through MMP-13-dependent invasion via the ERK1/2/AP-1 pathway. Carcinogenesis.

[12] Teicher B.A., Fricker S.P. (2010). CXCL12 (SDF-1)/CXCR4 pathway in cancer. Clin. Cancer Res..

[13] Xu C., Zhao H., Chen H., Yao Q. (2015). CXCR4 in breast cancer: Oncogenic role and therapeutic targeting. Drug Des. Devel. Ther..

[14] Xia J., Chen N., Hong Y., Chen X., Tao X., Cheng B., Huang Y. (2012). Expressions of CXCL12/CXCR4 in oral premalignant and malignant lesions. Mediat. Inflamm..

[15] Hong J.S., Pai H.K., Hong K.O., Kim M.A., Kim J.H., Lee J.I., Hong S.P., Hong S.D. (2009). CXCR-4 knockdown by small interfering RNA inhibits cell proliferation and invasion of oral squamous cell carcinoma cells. J. Oral Pathol. Med..

[16] Ishikawa T., Nakashiro K., Hara S., Klosek S.K., Li C., Shintani S., Hamakawa H. (2006). CXCR4 expression is associated with lymph-node metastasis of oral squamous cell carcinoma. Int. J. Oncol..

[17] Huang S.J., Tseng Y.K., Lo Y.H., Wu P.C., Lee J.H., Liou H.H., Liang C.C., Yang C.M., Wang C.C., Yen L.M. (2019). Association of SDF-1 and CXCR4 Polymorphisms With Susceptibility to Oral and Pharyngeal Squamous Cell Carcinoma. Anticancer Res..

[18] Taki M., Higashikawa K., Yoneda S., Ono S., Shigeishi H., Nagayama M., Kamata N. (2008). Up-regulation of stromal cell-derived factor-1alpha and its receptor CXCR4 expression accompanied with epithelial-mesenchymal transition in human oral squamous cell carcinoma. Oncol. Rep..

[19] Zhuang X.M., Zhou B. (2019). CXCR4 enhances cisplatin resistance of human tongue squamous cell carcinoma. J. Oral Pathol. Med..

[20] Petit I., Goichberg P., Spiegel A., Peled A., Brodie C., Seger R., Nagler A., Alon R., Lapidot T. (2005). Atypical PKC-zeta regulates SDF-1-mediated migration and development of human CD34+ progenitor cells. J. Clin. Investig..

[21] Huang S., Ouyang N., Lin L., Chen L., Wu W., Su F., Yao Y., Yao H. (2012). HGF-induced PKCzeta activation increases functional CXCR4 expression in human breast cancer cells. PLoS ONE.

[22] Basu A., Pal D. (2010). Two faces of protein kinase Cdelta: The contrasting roles of PKCdelta in cell survival and cell death. Sci. World J..

[23] Chen Z., Forman L.W., Williams R.M., Faller D.V. (2014). Protein kinase C-delta inactivation inhibits the proliferation and survival of cancer stem cells in culture and in vivo. BMC Cancer.

[24] Zhang N.H., Li J., Li Y., Zhang X.T., Liao W.T., Zhang J.Y., Li R., Luo R.C. (2012). Co-expression of CXCR4 and CD133 proteins is associated with poor prognosis in stage II-III colon cancer patients. Exp. Ther. Med..

[25] Miraglia S., Godfrey W., Yin A.H., Atkins K., Warnke R., Holden J.T., Bray R.A., Waller E.K., Buck D.W. (1997). A novel five-transmembrane hematopoietic stem cell antigen: Isolation, characterization, and molecular cloning. Blood.

[26] Jaszai J., Fargeas C.A., Florek M., Huttner W.B., Corbeil D. (2007). Focus on molecules: Prominin-1 (CD133). Exp. Eye Res..

[27] Singh S.K., Hawkins C., Clarke I.D., Squire J.A., Bayani J., Hide T., Henkelman R.M., Cusimano M.D., Dirks P.B. (2004). Identification of human brain tumour initiating cells. Nature.

[28] Horst D., Kriegl L., Engel J., Kirchner T., Jung A. (2008). CD133 expression is an independent prognostic marker for low survival in colorectal cancer. Br. J. Cancer.

[29] Chiou S.H., Yu C.C., Huang C.Y., Lin S.C., Liu C.J., Tsai T.H., Chou S.H., Chien C.S., Ku H.H., Lo J.F. (2008). Positive correlations of Oct-4 and Nanog in oral cancer stem-like cells and high-grade oral squamous cell carcinoma. Clin. Cancer Res..

[30] Ravindran G., Sawant S.S., Hague A., Kingsley K., Devaraj H. (2015). Association of differential beta-catenin expression with Oct-4 and Nanog in oral squamous cell carcinoma and their correlation with clinicopathological factors and prognosis. Head Neck.

[31] Zhang Q., Shi S., Yen Y., Brown J., Ta J.Q., Le A.D. (2010). A subpopulation of CD133(+) cancer stem-like cells characterized in human oral squamous cell carcinoma confer resistance to chemotherapy. Cancer Lett..

[32] Damek-Poprawa M., Volgina A., Korostoff J., Sollecito T.P., Brose M.S., O’Malley B.W., Akintoye S.O., DiRienzo J.M. (2011). Targeted inhibition of CD133+ cells in oral cancer cell lines. J. Dent. Res..

[33] Zhang S.S., Han Z.P., Jing Y.Y., Tao S.F., Li T.J., Wang H., Wang Y., Li R., Yang Y., Zhao X. (2012). CD133(+)CXCR4(+) colon cancer cells exhibit metastatic potential and predict poor prognosis of patients. BMC Med..

[34] Hermann P.C., Huber S.L., Herrler T., Aicher A., Ellwart J.W., Guba M., Bruns C.J., Heeschen C. (2007). Distinct populations of cancer stem cells determine tumor growth and metastatic activity in human pancreatic cancer. Cell Stem Cell.

[35] Yu C.C., Hu F.W., Yu C.H., Chou M.Y. (2016). Targeting CD133 in the enhancement of chemosensitivity in oral squamous cell carcinoma-derived side population cancer stem cells. Head Neck.

[36] Pindborg J.J., Wahi P.N. (1997). Histological Typing of Cancer and Precancer of the Oral Mucosa.

[37] Ravindran G., Devaraj H. (2015). Prognostic significance of neural stem cell markers, Nestin and Musashi-1, in oral squamous cell carcinoma: Expression pattern of Nestin in the precancerous stages of oral squamous epithelium. Clin. Oral. Investig..

[38] Moitra K. (2015). Overcoming Multidrug Resistance in Cancer Stem Cells. Biomed. Res. Int..

[39] Uchida D., Begum N.M., Almofti A., Nakashiro K., Kawamata H., Tateishi Y., Hamakawa H., Yoshida H., Sato M. (2003). Possible role of stromal-cell-derived factor-1/CXCR4 signaling on lymph node metastasis of oral squamous cell carcinoma. Exp. Cell Res..

[40] Delilbasi C.B., Okura M., Iida S., Kogo M. (2004). Investigation of CXCR4 in squamous cell carcinoma of the tongue. Oral Oncol..

[41] Takabayashi T., Takahashi N., Okamoto M., Yagi H., Sato M., Fujieda S. (2009). Lipopolysaccharides increase the amount of CXCR4, and modulate the morphology and invasive activity of oral cancer cells in a CXCL12-dependent manner. Oral Oncol..

[42] Lee J.I., Jin B.H., Kim M.A., Yoon H.J., Hong S.P., Hong S.D. (2009). Prognostic significance of CXCR-4 expression in oral squamous cell carcinoma. Oral Surg. Oral Med. Oral Pathol. Oral Radiol. Endod..

[43] Lu C.L., Ji Y., Ge D., Guo J., Ding J.Y. (2011). The expression of CXCR4 and its relationship with matrix metalloproteinase-9/vascular endothelial growth factor in esophageal squamous cell cancer. Dis. Esophagus.

[44] Uchida D., Onoue T., Kuribayashi N., Tomizuka Y., Tamatani T., Nagai H., Miyamoto Y. (2011). Blockade of CXCR4 in oral squamous cell carcinoma inhibits lymph node metastases. Eur. J. Cancer.

[45] Ravindran G., Devaraj H. (2012). Aberrant expression of CD133 and musashi-1 in preneoplastic and neoplastic human oral squamous epithelium and their correlation with clinicopathological factors. Head Neck.

[46] Lu C., Xu F., Gu J., Yuan Y., Zhao G., Yu X., Ge D. (2015). Clinical and biological significance of stem-like CD133(+)CXCR4(+) cells in esophageal squamous cell carcinoma. J. Thorac. Cardiovasc. Surg..

