# Supplementary material for: Stem Cell Markers CXCR-4 and CD133 Predict Aggressive Phenotype and Their Double Positivity Indicates Poor Prognosis of Oral Squamous Cell Carcinoma
Source: Cancers (Basel). 2021 Nov 23;13(23):5895. doi: 10.3390/cancers13235895 (PMC8656999; doi:10.3390/cancers13235895)
Supplement: Supplementary file 1 [file cancers-13-05895-s001.zip › cancers-1420806-supplementary.pdf]

Supplementary Material: Stem Cell Markers CXCR-4 and CD133 Predict Aggressive Phenotype and Their Double Positivity Indicates Poor Prognosis of Oral Squamous Cell Carcinoma

Ravindran Caspa Gokulan and Halagowder Devaraj

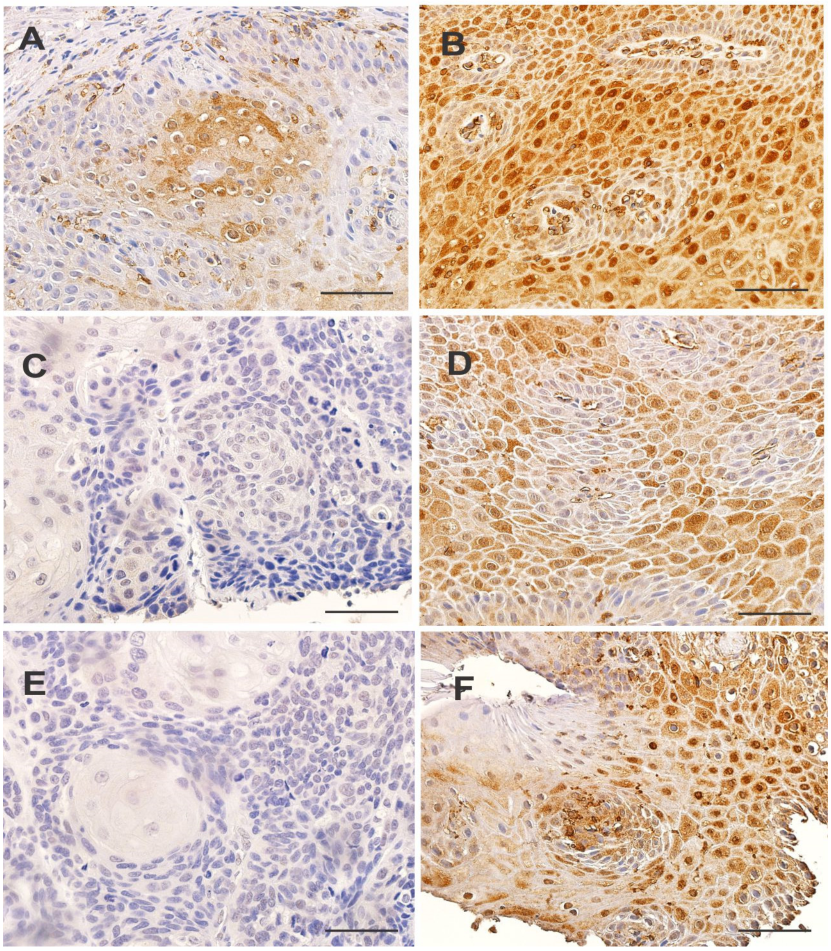

Figure S1. Representative images of Immunostaining of CXCR-4 (A & B), PKC-δ (C & D) and CD133 (E & F) in oral squamous cell carcinoma tissues. The representative images of negative score was provided in the left panel and positive score was provided in the right panel. Scale bar = 20μm.

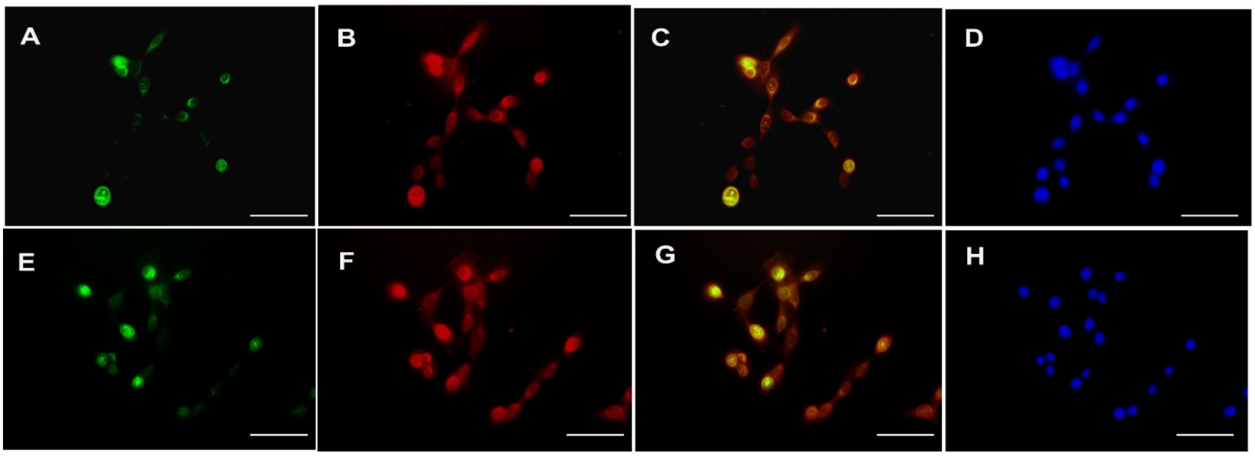

Figure S2. Double Immunofluorescence analysis of CXCR-4 with PKC-δ and CD133 in human esophageal cancer cell line CAL27. Upper panel: CXCR4 (A), PKC-δ (B), CXCR-4 and PKC-δ co-localization (C) and DAPI (D) staining in CAL27 cells. Lower panel: CXCR4 (E), CD133 (F), CXCR-4 and CD133 co-localization (G) and DAPI (H) staining in CAL27 cells. (Scale bar - 20μm).

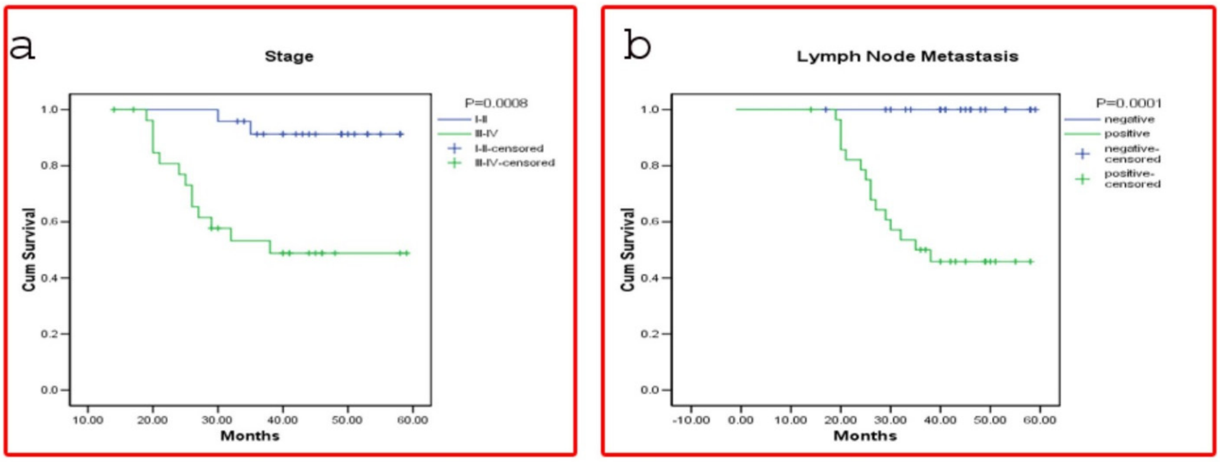

Figure S3. Overall survival of patients with oral squamous cell carcinoma according to Stage (p=0.0008) (a) and Lymph node metastasis (p=0.0001) (b) calculated by the Kaplan-Meier method.

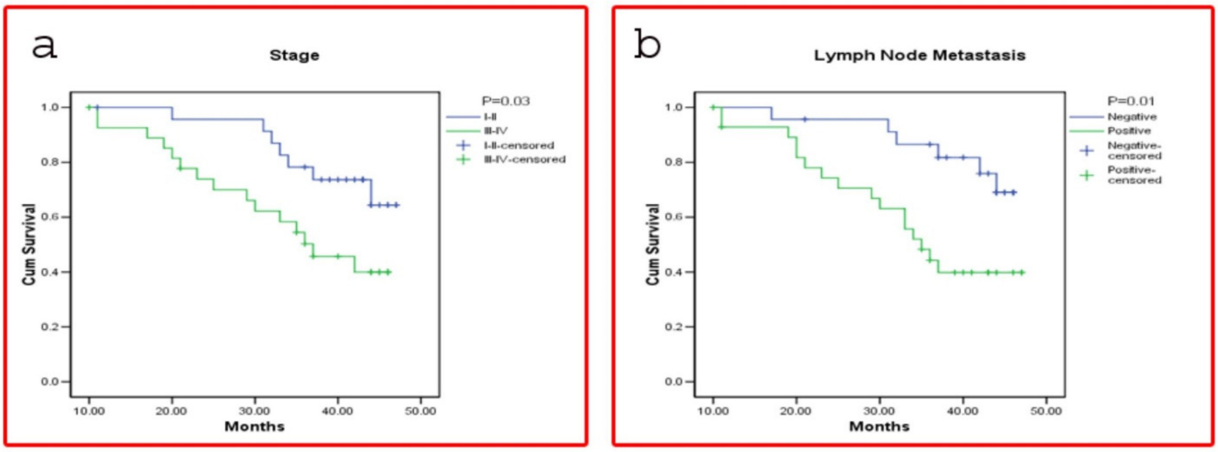

Figure S4. Disease-free survival of patients with oral squamous cell carcinoma according to Stage (p=0.03) (a) and Lymph node metastasis (p=0.01) (b) calculated by the Kaplan-Meier method.
